# Phytochemical Analysis and *In Vitro* Antioxidant and Antibacterial Activity of Different Solvent Extracts of *Beilschmiedia roxburghiana* Nees Stem Barks

**DOI:** 10.1155/2022/6717012

**Published:** 2022-03-26

**Authors:** Lekha Nath Khanal, Khaga Raj Sharma, Yuba Raj Pokharel, Surya Kant Kalauni

**Affiliations:** ^1^Central Department of Chemistry, Tribhuvan University, Kathmandu, Nepal; ^2^Department of Chemistry, Prithvi Narayan Campus, Tribhuvan University, Pokhara, Nepal; ^3^Faculty of Life Science and Biotechnology, South Asian University, New Delhi, India

## Abstract

Plants have long been considered as a basis of medicines for different indigenous cultures around the globe. They continue as a prominent source of important phytoconstituents which exhibit significant biological activities. In this study, we performed the phytochemical screening, estimation of total phenolic and flavonoids, antioxidants, and antimicrobial activities of the stem bark of *Beilschmiedia roxburghiana* Nees using different solvents. The total phenolic and total flavonoid contents ranged from 106.73 ± 1.62 mg GAE/g and 99.32 ± 0.66 mg QE/g (methanol extract) to 65.59 ± 1.79 mg GAE/g and 29.98 ± 0.90 mg QE/g (n-hexane extract), respectively. The maximum 2,2-diphenyl-1-picrylhydrazyl (DPPH) free radical scavenging activity with a half-maximal inhibitory concentration (IC_50_) of 39.86 ± 3.69 *μ*g/mL was observed for methanol extract followed by aqueous (IC_50_ = 43.55 ± 6.16 *μ*g/mL), ethyl acetate (IC_50_ = 44.30 ± 5.88 *μ*g/mL), dichloromethane (IC_50_ = 71.50 ± 4.70 *μ*g/mL), and the lowest activity was observed for n-hexane extract. The disc diffusion method revealed that the ethyl acetate extract exhibited relatively higher activity against *Salmonella typhi* (ZOI = 13 mm), and moderate activities against *Shigella sonnei, Acinetobacter baumannii*, *Klebsiella pneumoniae,* and *Staphylococcus aureus* (ZOI = 12 mm). The methanol and aqueous extracts showed nearly parallel and the n-hexane and dichloromethane extracts exhibited mild antibacterial activities. The results indicated that the polarity index of the extracting solvents amplified the biological activities of the extract. The study is helpful to support the validity of the traditional application of the plant as natural medicine.

## 1. Introduction

Medicinal plants, microorganisms, and animals are the leading sources of traditional medicines against different illnesses over many centuries. The plant-derived compounds share a major portion of medicines that have been used by humans against several diseases. The global market of medicine that worth about 1.1 trillion US dollars, comprises of plant-based medicines about 25%, microorganisms 13%, and animals 3% [1]. Especially, people residing in developing countries rely on natural products as a major source of medicines due to their low cost, fewer side effects, and availability [2]. The use of herbal medicines continuously plays a vital role in primary health care, and about 80% of the world's population is estimated to depend on traditional medicine for their primary healthcare [3]. The development of knowledge of the exercise and consciousness of medicinal plants by different human civilizations in the past has augmented the notions and capacity of pharmacists and physicians to tackle the challenges of spreading professional services to assist human health [4].

Under certain conditions, reactive oxygen species (ROS) and reactive nitrogen species (RNS) are produced in the living cells during mitochondrial respiration. These free radicals which are more reactive than molecular oxygen cause oxidative injuries on biomacromolecules such as proteins, nucleic acids, and lipids [5]. When our natural antioxidant defense and repair system is incompetent to alleviate them, a state of oxidative stress is set up leading to adverse effects such as DNA damage, cellular degeneration, carcinogenesis, and aging. This condition is responsible for various ailments such as Alzheimer's disease, Parkinson's disease, cancer, epilepsy, inflammation, retrolental fibroplasia, atherosclerosis, ischemia-reperfusion, lung injury, and other disorders [6]. The concentrations of the free radicals in our body are controlled by the endogenous antioxidant arrangement as well as exogenous nutrient supplements. They protect from apparent intracellular and extracellular oxidative stress. Antioxidants in the diet can delay or hinder lipid oxidation, react with reactive oxygen and nitrogen species and alleviate cellular damage by terminating the detrimental chain reactions [7].

Many valuable secondary metabolites such as alkaloids, flavonoids, phenolics, terpenoids, tannins, essential oils, and coumarins, etc. exhibit awesome antibacterial activities. These compounds are used as precursors for the synthesis of valuable antibiotics to treat several infections in the skin, urinary, gastrointestinal, and respiratory systems [8, 9]. Despite the development of a large number of antibiotics against several infectious diseases, the microbial resistance is continuously increasing all over the world [10]. The speedy rise of multidrug-resistant (MDR) bacteria is constantly pressurizing the global healthcare system for a few decades. Plants contribute to a large chemodiversity of active compounds which provide a hopeful source of antibacterial lead compounds to develop effective drugs against antibiotic-resistant bacteria [11].

Plants are the big reservoirs of important secondary metabolites but they need proper solvents of matching polarity for efficient extraction. The documentation of the active extracts or fractions is crucial for future research on the isolation and pharmaceutical application of significant compounds [12]. There are several noteworthy compounds isolated from plants such as cocaine, digitoxin, quinine, morphine, and codeine which are still used as drugs. The exploration of new compounds from a natural source is exciting in present and future times for the sustainable conservation and utilization of biodiversity. The progress in plant tissue culture, sophisticated extraction, isolation and characterization tools, and modern biotechnological approaches have simplified the production and standardization of herbal medicines, to elucidate analytical marker compounds [13, 14]. Natural products such as prostaglandins, steroids, and peptides hormones play a vital role in the pharmaceutical industries to boost the inspiration for drug discovery during the 20^th^ century [15].

*Beilschmiedia* is a pantropical genus of the Lauraceae family that contains about 250 species distributed in Asia and Africa. *B. roxburghiana* Nees is a medium-sized evergreen tree growing in tropical forests of China, India, and Myanmar. It bears alternate elongated leaves which are petiolate, and pinnately veined. Flowers are small, bisexual, and greenish with six tepals that develop into an ellipsoid, pyriform, or spherical fruits [16, 17]. In Southeast Asia, the plant is abundant in the subtropical forest at the altitudes of 200–400 m and is commonly used for treating bone-related problems like arthritis, renal problems, rheumatism, and as timber in Bhutan [18, 19]. The plants of this genus have been extensively studied in the past decades because of their widespread application in traditional medicine. These investigations had led to the isolation of important secondary metabolites like endiandric acid derivatives, epoxyfuranoid lignans, kingianins, and alkaloids having significant antibacterial, anti-inflammatory, enzyme inhibitory, and anticancer activities [20].

In the Kaski district of Nepal, the plant is known as “Hadchur” and local people use its stem bark in bone injuries and fractures. To the best of our knowledge, it is the first scientific study of the plant for phytochemical and biological activities. This research is aimed at the phytochemical screening, estimation of total phenolics and flavonoids, antioxidants, and antibacterial activities of extracts of the plant using water, methanol, ethyl acetate, dichloromethane, and *n*-hexane as solvents. The results of the study might be supportive to validate the traditional application of the plant as natural medicine.

## 2. Materials and Methods

### 2.1. Chemicals

All the chemicals and reagents of the highest purity and distilled water (DW) were used throughout the lab works. The Folin–Ciocalteu reagent (FCR), AlCl_3_, HCl, Na_2_CO_3_, H_2_SO_4_, CH_3_COOK, and dimethyl sulphoxide (DMSO) were purchased from Thermo Fisher Scientific India, Pvt. Ltd. Gallic acid, ascorbic acid, Muller Hinton Agar (MHA), Muller Hinton Broth (MHB), and quercetin of Himedia Laboratories Company Ltd., India, were used. Similarly, ethyl acetate, methanol, ethanol, chloroform, n-hexane, and dichloromethane from Merck Life Science Limited were used. Neomycin and 2,2-diphenyl-1-picrylhydrazyl (DPPH) were obtained from Sigma-Aldrich and Tokyo Chemical industries Co. Ltd, respectively.

### 2.2. Plant Collection and Extraction

The stem barks of the plant were collected from Syastri, village of Kaski district of Nepal, in July 2020. The herbarium was submitted to the National Herbarium and Plant Laboratories, Lalitpur, Nepal, to get the botanical identification (voucher No L20). The shade-dried samples were pulverized using a mechanical grinder. The dry powder (50 gm in 500 mL) was dipped for 7 days into methanol, ethyl acetate, dichloromethane, n-hexane, and water separately. The extracts were filtered, concentrated using a rotary evaporator (IKA RV10), and stored in a refrigerator for use.

### 2.3. Phytochemical Screening

The presence of diverse phytochemicals in different extracts of the bark of *B. roxburghiana* was assessed by adopting standard protocols [21–23]. Tests were performed for the presence of alkaloids, anthracenes, carotenes, coumarin, flavonoids, glycosides, phytosterols, polyphenols, reducing sugars, saponins, tannins, and terpenoids.

### 2.4. Estimation of Total Phenolic Content (TPC) and Total Flavonoid Content (TFC)

The quantification of TPC in different extracts of *B. roxburghiana* was performed by the Folin–Ciocalteu method with a slight change [24, 25]. A set of standard gallic acid solutions of 5, 10, 20, 40, 50, and 60 *μ*g/mL were prepared. Aliquots of 20 *μ*L of the gallic acid and the test solutions (5 mg/mL) were filled in the bores of a microplate in triplicates. To each of the solutions, 100 *μ*L of FCR (1 : 10 in DW) and 80 *μ*L of 7.5% Na_2_CO_3_ solution were added. The mixture was incubated for 25 minutes in dark at room temperature, and optical density was recorded at 765 nm using a microplate reader (Biotek Multimode reader). The TPC was calculated from the standard calibration curve and expressed as milligrams gallic acid equivalents per gram (mg GAE/g) of the dry extract.

The TFC in the extracts of different solvents of *B. roxburghiana* stem bark was determined by the aluminum chloride colorimetric method [25, 26] with slight adjustments. The standard quercetin solutions of 10, 20, 30, 40, 50, 60, and 80 *μ*g/mL were prepared in methanol. The aliquots of 130 *μ*L of quercetin, 5 *μ*L of AlCl_3_, 5 *μ*L of CH_3_COOK, and 60 *μ*L of ethanol were loaded into a 96-well microplate in triplicates. Similarly, 20 *μ*L of each of the extracts (5 mg/mL), 110 *μ*L of double-distilled water, 5 *μ*L of AlCl_3_, 5 *μ*L of CH_3_COOK, and 60 *μ*L of ethanol were also filled in triplicates. The microplate was incubated for 30 minutes in the dark, and the absorbance was recorded at 415 nm against the blank. The TFC was calculated from the standard curve and expressed as milligrams quercetin equivalent per gram (mg QE/g) of the dry extract.

### 2.5. In Vitro Antioxidant Activity

The antioxidant activity of different extracts of the plant was evaluated by the DPPH free radical scavenging method [27, 28] with slight modifications. The test solutions of the extracts and ascorbic acid of 250, 125, 62.5, 31.25, 15.6, and 7.8 *μ*g/mL were prepared in 50% DMSO. Each of 100 *μ*L 0.1 mM DPPH and test solutions were loaded into a 96-well microplate in triplicates. Similarly, ascorbic acid was loaded as a positive control. The mixture was incubated for 30 minutes in dark at lab temperature, and the optical density was recorded at 517 nm against blank. The data were processed and analyzed by using Microsoft Excel 2016. The radical scavenging activity of different extracts and ascorbic acid was calculated:

(1) $$\begin{array}{c} \text{Percentage scavenging}=\frac{\text{The absorbance of control}-\text{absorbance of sample}}{\text{Absorbance of control }}\times 100. \end{array}$$

The half-maximal inhibitory concentration (IC_50_) of the extracts and the positive controls were calculated by using the Graph Pad Prism 9 software.

### 2.6. Antimicrobial activity

The antibacterial activity of different extracts of *B. roxburghiana* was evaluated by the agar well diffusion method [29, 30]. The test was performed against five Gram-negative and one Gram-positive bacteria. Six samples of the American Type Culture Collection (ATCC) bacteria were subcultured on the Muller Hinton Agar (MHA) from the pure samples and stored at 4°C.  Table 1 shows the list of bacteria used in the study. The microorganisms were grown up in the freshly prepared Muller Hinton Broth (MHB) solution overnight at 37°C to equate turbidity to 0.5 McFarland's standard. The bacterial suspension was carpet-cultured on the sterile Muller Hinton Agar (MHA) media with cotton swabs. The boreholes of 6 mm diameter were punched at equivalent distances on the surface by using a sterile cork-borer.

The wells were fed with 50 *μ*L of the plant extracts (50 mg/mL), neomycin (1 mg/mL), and 50% DMSO as a control. The plates were incubated for 24 hours at 37°C. On the next day, the samples were taken out from the incubator and the clear zones around the holes that correspond to the respective inhibition zones were measured and recorded.

## 3. Results and Discussion

### 3.1. Phytochemical Screening

The different extracts of the plant showed the presence of phytochemicals. Out of the 12 phytochemicals screened, BRM showed the abundance of 11 phytochemicals followed by BRE and BRA with 10 phytochemicals. Six and five phytochemicals were detected in BRD and BRH, respectively. All the extracts contained alkaloids, flavonoids, polyphenols, and tannins (Table 2). The polarity of solvents is found proportional to the presence of phytochemicals. The more polar solvents such as methanol, ethyl acetate, and water extracts picked up the phytochemicals more efficiently than DCM and n-hexane. Thilagavathi et al. [31] also reported the same trend of the abundance of phytochemicals in different extracting solvents. The polarity index of the solvents, time, and method of extraction greatly influence the quality and quantity of phytochemicals in plants [12]. The secondary metabolites of plants are significant compounds that exhibit specific biological and pharmacological activities. The phytochemicals such as alkaloids, flavonoids, carbohydrates, polyphenols, steroids, and terpenoids have diverse physiological actions in the human and animal body.

So, they have a substantial role in the development of natural drugs against different health complications [32].

### 3.2. Total Phenolic and Total Flavonoid Content

The results of TPC and TFC contents among different extracts are presented in Table 3. The BRM showed the highest phenolic (106.73 ± 1.62 mg GAE/g) and flavonoid (99.32 ± 0.66 mg·QE/g) contents, and the lowest values were obtained for BRH (TPC = 65.59 ± 1.79 mg·GAE/g, TFC = 29.98 ± 0.90 mg·QE/g). Similarly, the BRA (TPC = 78.41 ± 0.34 mg·GAE/g, TFC = 39.86 ± 0.90 mg·QE/g), BRE (TPC = 90.69 ± 2.71 mg·GAE/g, TFC = 49.79 ± 1.07 mg·QE/g), and BRD (TPC = 74.87 ± 0.93 mg·GAE/g), TFC = 35.59 ± 0.90 mg·QE/g) were obtained. The TPC and TFC values are influenced by the composition and polarity of the solvent system. Similar types of variation of TPC and TFC in the extracts of different solvents was reported by Do et al. [33] in *Limnophila aromatica*. An Indian traditional plant, *Meyna spinosa* Roxb. Ex Link, was reported to have TPC and TFC values in the increasing order of polarity of the solvents in the order of methanol > ethyl acetate > petroleum ether [34].

### 3.3. In Vitro Antioxidant Activity

**Figure 1:**
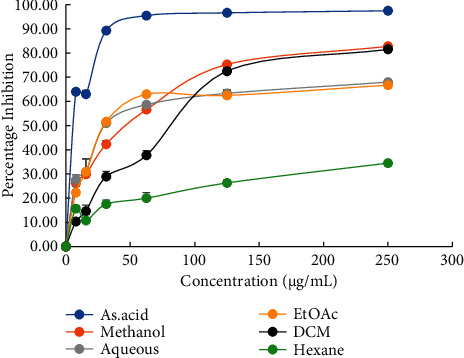
DPPH radical inhibiting capacity of different extracts of *B. roxburghiana*.

The DPPH method is short, efficient, and used extensively to predict the antioxidant capacity of plant extracts. In this technique, the violet color of the DPPH solution is reduced to yellow on the addition of the extract in a dose-dependent manner. The polyphenols and tocopherols in the extract scavenge the DPPH radical due to their hydrogen donating ability [35]. *B. roxburghiana* stem bark extracts of different solvents showed a linear concentration-response relationship to DPPH radical inhibition capacity. The increase in the concentration of the extracts was proportional to the corresponding scavenging capacity as shown in Figure 1. Ascorbic acid showed the highest scavenging capacity with an IC_50_ value of 6.40 ± 0.29 *μ*g/mL. Table 3 shows that the IC_50_ value of BRM (39.86 ± 3.69 *μ*g/mL) was followed by the BRA (43.55 ± 6.16 *μ*g/mL), BRE (44.30 ± 5.88 *μ*g/mL), and BRD (71.50 ± 4.70 *μ*g/mL). The lowest antioxidant activity was shown by the BRH with a very high IC_50_ value of (1498.67 ± 62.13 *μ*g/mL). Our results showed that the extracts having relatively high TPC and TFC showed better scavenging power. Like the results of Do et al. [33]; we observed that the methanol, ethyl acetate, and water which can catch up with the phytoconstituents efficiently had relatively lower IC_50_ values than that of less polar BRH and BRD. Sen et al. [34] also reported a similar trend of variation of antioxidant capacity which was linearly dependent upon the TPC and TPC of the extracts.

Antioxidants are the key constituents that defend our bodies from the damages caused by free radical-induced oxidative stress. Plants provide many important compounds which offer resistance against oxidative stress by scavenging free radicals, preventing lipid peroxidation, and other mechanisms. The micronutrients such as vitamin C and E, *β*-carotene, and other important ingredients such as phenolic and flavonoids from plants are helpful to reduce oxidative stress [34]. The present study was undertaken to compare the antioxidant activity of *B. roxburghiana* stem bark by using solvents of different polarities. The results of this study indicate that the extracts of the plant showed radical scavenging activity due to their electron transfer or hydrogen donating ability because the total phenolic and total flavonoid contents in plants are proportional to the radical scavenging activity.

### 3.4. Antibacterial Activity

The antibacterial activity of different extracts of the *B. roxburghiana* against the tested microorganisms is given in Table 4.

**Figure 2:**
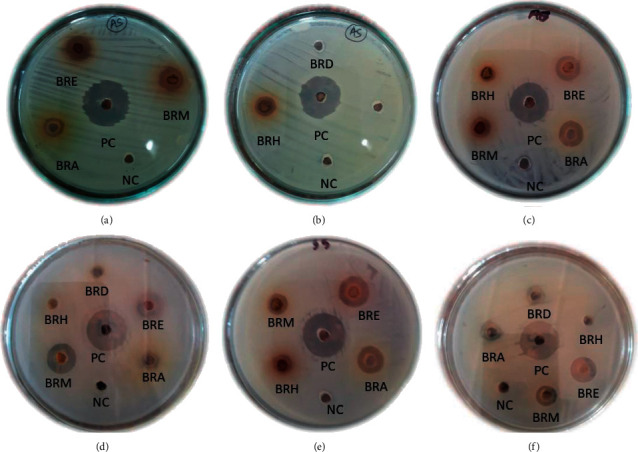
Antibacterial slides: (a, b) *S. aureus*, (c) *A.* baumannii, (d) *S. sonnei*, (e) *S. typhi*, and (f) *K. pneumoniae*. PC, positive control; NC, negative control; BRA, aqueous extract; BRE, ethyl acetate extract; BRH, n-hexane extract; BRD, DCM extract; and BRM, methanol extract.

The plant showed a diverse susceptibility towards the tested bacteria (Figure 2). All the extracts were found ineffective towards *E. coli* and moderate towards other organisms. The BRH against *K. pneumoniae*, *S*. *sonnei*, and *A. baumannii* as well as BRD against *S*. *aureus* and *S. sonnei* were ineffective. The BRE exhibited the highest activity against *S. typhi* (ZOI = 13 mm), followed by *K. pneumoniae*, *S. aureus, S. sonnei*, and *A. baumannii* (ZOI = 12 mm). The methanol and aqueous extracts showed a parallel activity towards the tested bacteria. The BRM showed the highest activity against *S. sonnie* (ZOI = 13 mm) followed by *S. aureus* (ZOI = 12), *S. typhi* (ZOI = 11 mm), and *K. pneumonia* (ZOI = 10), and minor activity against *A. baumannii* (ZOI = 9 mm). Similarly, the BRA exhibited the highest activity against *S. typhi* and *A. baumannii* (ZOI = 12 mm) followed by *S. sonnei* (ZOI = 11 mm) and *S. aureus* (ZOI = 10 mm), and the lowest activity was shown against *K. pneumoniae* (ZOI = 8 mm).

The antibacterial activity of endiandric acid derivatives (beilschmiedic acids A, B, and C) isolated from the stem bark of *B. anacardioides* from Cameroon was evaluated by the agar well diffusion method. The compounds were inactive against *E. coli* and *Pseudomonas palida* but active against *Bacillus subtilis, Micrococcus luteus,* and *Streptococcus faecalis* in terms of the zone of inhibition (ZOI) and minimum inhibitory concentration (MIC) values. Beilschmiedic acid C showed the highest activity against *M. luteus* (ZOI = 30 mm, MIC = <0.7) followed by *B. subtilis* (ZOI = 13 mm, MIC = 5.6 *μ*M) and *S. faecalis* (ZOI = 18 mm, MIC = 22.7 *μ*M). This result is comparable to our results of inactivity against *E. coli* [36]. The endiandric acid derivatives might be present in the stem bark of *B. roxburghiana* which are passive towards some of the bacteria including *E. coli* and *P. palida.* The methanol stem bark extracts of *B. acuta* were found active against the 26 tested Gram-negative bacteria with MIC values ranging from below 8 to 256 *μ*g/mL [37]. Salleh et al. [38] determined the antibacterial activities of the leaves and stem bark extracts of ethyl acetate, methanol, and n-hexane of *B. madang*, *B. glabra,* and *B. pulverulenta* from Malaysia. The extracts were evaluated for the MIC and minimum bactericidal concentration (MBC) against three Gram-positive: *B. subtilis* (ATCC 6633), *S. aureus* (ATCC 29737), and *Enterococcus faecalis* (ATCC 19433), and three Gram-negative: *Pseudomonas aeruginosa* (ATCC 9027), *E. coli* (ATCC 10536), and *K. pneumoniae* (ATCC 13883) bacteria by microdilution method. The extracts exhibited weak to moderate activity with MIC and MBC values ranging from 125 to 1000 *μ*g/mL. Most of the extracts showed weak activities against the Gram-negative bacteria in comparison to that of Gram-positive bacteria which might be due to their thick cell wall. Extensive research is necessary to identify and isolate the active compounds from *B. roxburghiana* responsible for substantial biological properties. So, the plant might be a source of natural antioxidants and antibiotics for the food industries to replace the synthetic chemicals.

## 4. Conclusions

This paper is the first-time report of a comprehensive study on phytochemical screening, estimation of total phenolics and flavonoids, antioxidants, and antimicrobial activities of *B. roxburghiana* of Nepalese origin. The secondary metabolites in plants require suitable solvents of the specific polarity index for the extraction. The identification of active extracts or fractions is helpful for the fundamental knowledge of pharmaceutical application and the isolation of active compounds. The antioxidant and antibacterial activities of the extracts were dependent upon the relative proportion of TPC and TFC. The polar solvents such as methanol, water, and ethyl acetate efficiently picked up the polar phenolic and flavonoid compounds from the plant material. Extensive research is warranted for the evaluation of different biological properties, bioassay-guided isolation of important secondary metabolites, their pharmacology, safety, clinical trials, and product development. It can strengthen the use of the plant as a vital resource of natural biodiversity for the sustainable development of novel potential therapeutic drugs or lead compounds.

## Figures and Tables

**Table 1:** List of bacterial strains used for the study.

Microorganisms	Type	ATCC
*Acinetobacter baumannii*	Gram-negative	19606
*Shigella sonnei*	Gram-negative	25931
*Klebsiella pneumoniae*	Gram-negative	700603
*Staphylococcus aureus*	Gram-positive	25923
*Escherichia coli*	Gram-negative	25922
*Salmonella typhi*	Gram-negative	14028

**Table 2:** Results of phytochemical screening.

S. No	Secondary metabolites	BRM	BRE	BRA	BRD	BRH
1	Alkaloids	++	+	++	+	++
2	Flavonoids	+	++	++	+	+
3	Carotene	+	+	+	+	−
4	Polyphenols	++	++	++	++	+
5	Glycosides	+	+	+	−	−
6	Terpenoids	++	+	+	−	−
7	Saponins	++	+	+	−	−
8	Tannins	++	++	+	+	+
9	Coumarins	+	−	−	−	−
10	Anthracene	+	+	−	+	+
11	Reducing sugar	−	−	+	−	−
12	Phytosterol	++	++	++	+	−

BRM, methanol extract; BRE, ethyl acetate extract; BRA, aqueous extract; BRD, dichloromethane extract; BRH, n-hexane extract; +, present; −, absent.

**Table 3:** TPC, TFC, and antioxidant activity (IC_50_) of different extracts of *B. roxburghiana*.

Extracts	TPC (mg GAE/g)	TFC (mg QE/g)	IC_50_ (*μ*g/mL)
BRA	78.41 ± 0.34	39.86 ± 0.90	43.55 ± 6.16
BRE	90.69 ± 2.71	49.79 ± 1.07	44.30 ± 5.88
BRM	106.73 ± 1.62	99.32 ± 0.66	39.86 ± 3.69
BRD	74.87 ± 0.93	35.59 ± 0.90	71.50 ± 4.70
BRH	65.59 ± 1.79	29.98 ± 0.90	1498.67 ± 62.13
^*∗*^Ascorbic acid	—	—	6.40 ± 0.29

Values are the mean ± SD (*n* = 3), ^*∗*^Positive control.

**Table 4:** Antibacterial activity of different extracts of *B. roxburghiana*.

Zone of inhibition (ZOI) in mm						
Microorganisms	BRH	BRD	BRE	BRM	BRA	^*∗*^Neomycin
*Klebsiella pneumoniae*	—	7	12	10	8	18
*Escherichia coli*	—	—	—	—	—	17
*Salmonella typhi*	7	Nd	13	11	12	18
*Staphylococcus aureus*	9	—	12	12	10	22
*Shigella sonnei*	—	—	12	13	11	19
*Acinetobacter baumannii*	—	Nd	12	9	12	18

## Data Availability

The data used to support the findings of the study are available from the corresponding author.
